# Low Back Pain in Korea: Survey Weighted Analysis with Age Sex and Lumbar Radiographic Grade Matching

**DOI:** 10.3390/healthcare14040422

**Published:** 2026-02-07

**Authors:** Taewook Kim

**Affiliations:** Department of Orthopedic Surgery, Seoul National University Hospital, Seoul 03080, Republic of Korea; ray0601@snu.ac.kr

**Keywords:** low back pain, epidemiology, Korea, propensity score matching, lumbar X-ray

## Abstract

**Highlights:**

**What are the main findings?**

- In a nationally representative sample of Korean adults aged 50–79 years (KNHANES 2010–2011; weighted estimate 8,464,167), 25.1% reported low back pain (LBP) and 28.3% had radiographic lumbar spondylosis (Kellgren–Lawrence grade ≥ 2).
- Even under comparable objective conditions (matched by age, sex, and lumbar X-ray grade), LBP showed consistent associations with depressive mood (≥2 weeks), osteoporosis, lower household income, higher alcohol intake and physical activity, with additional sex-specific patterns in metabolic and behavioral factors.

**What are the implications of the main findings?**

- LBP in Korea appears to be influenced by psychosocial, socioeconomic, and systemic health factors beyond lumbar radiographic severity, supporting a more holistic risk assessment rather than reliance on imaging alone.
- Several psychosocial, socioeconomic, and systemic health factors were observed to co-occur with LBP beyond lumbar radiographic severity, highlighting the multidimensional nature of LBP in older adults.

**Abstract:**

**Background/Objectives**: Low back pain (LBP) is a major public health problem that contributes substantially to disability and impaired quality of life. **Methods**: Using nationally representative data from the fifth Korea National Health and Nutrition Examination Survey (KNHANES V), this study evaluated the epidemiology of LBP among Korean adults aged 50–79 years and explored factors associated with LBP in relation to lumbar radiographic findings. Weighted analyses corresponding to an estimated 8,464,167 individuals were performed. Multivariable logistic regression was used to explore factors associated with LBP. In addition, to explore patterns of association under comparable demographic and radiographic conditions, we conducted analyses stratified by age, sex, and lumbar radiographic grade as a descriptive, exploratory approach. **Results**: In weighted estimates, 25.1% of participants reported LBP and 28.3% demonstrated radiographic lumbar spondylosis (Kellgren–Lawrence grade ≥ 2). LBP was consistently associated with depressive mood, osteoporosis, lower household income, and lifestyle-related factors, even under similar age and radiographic conditions. **Conclusions**: These findings suggest that LBP in Korea may reflect multidimensional health factors beyond lumbar radiographic severity alone.

## 1. Introduction

Low back pain (LBP) stands as a significant global health challenge, manifesting through discomfort, muscle tension, or stiffness between the costal margin and the inferior gluteal folds, often extending to leg pain [1,2]. The essential role of the spine in balance and mobility means that LBP can severely impair daily functioning and diminish quality of life [3].

Despite a variety of treatment options, the burden of LBP is escalating, with recent estimates suggesting around 245.9 million new cases each year. This accounts for 3.2% of global incidents, placing LBP as the 15th leading cause worldwide of disability-adjusted life years (DALYs) in 2020—a staggering increase of nearly 50% in two decades [4,5]. These statistics underscore the growing health and social impact of LBP, highlighting the urgent need for research focused on the early detection and prevention of this condition, especially in high-risk populations.

Epidemiological studies have consistently shown that the prevalence and burden of LBP vary by age, sex, and socioeconomic context, and that radiographic degenerative changes in the spine often show limited concordance with pain symptoms [6]. Previous population-based studies have reported that a substantial proportion of individuals with radiographic degeneration are asymptomatic, whereas many patients with clinically significant LBP demonstrate minimal abnormalities on imaging [6]. These observations highlight the importance of evaluating LBP beyond structural findings alone.

Notably, research on LBP has often overlooked the comprehensive analysis of participant characteristics that could inform early detection and risk mitigation strategies [7]. This gap prompted us to undertake this study using nationally representative health survey data from the Korea National Health and Nutrition Examination Survey (KNHANES). By incorporating the complex sampling design and survey weights, our study provides an opportunity to describe the epidemiology of LBP and its associated factors at a national scale among Korean adults aged 50–79 years.

This research utilizes data from the fifth KNHANES, a dataset characterized by its rigorous sampling methodology that is designed to generate nationally representative health estimates for the Korean population. This approach enables an in-depth analysis across a broad spectrum of demographics and socioeconomic indicators, using survey-weighted estimates corresponding to 8,464,167 individuals [8].

By examining associated factors under comparable objective conditions—specifically, among individuals of the same age, sex, and Kellgren–Lawrence (K-L) grade—we aim to provide detailed insights that can guide the early detection and management of LBP. This targeted approach has the potential to significantly contribute to our understanding of LBP, informing both clinical practice and public health strategies aimed at mitigating this condition’s impact. Through this research, we seek to advance the field’s knowledge of LBP, particularly within the Korean context, which may hold unique demographic or health-related dynamics influencing the prevalence and management of LBP.

Unlike prior epidemiologic studies that primarily focused on symptom prevalence or imaging findings in isolation, this study examines LBP under objectively comparable demographic and radiographic conditions using nationally representative survey data. By evaluating factors associated with LBP among individuals of similar age, sex, and lumbar radiographic grade, this study aims to explore correlates of LBP beyond conventional imaging-based explanations.

## 2. Materials and Methods

### 2.1. Data Source

Our research included participants from KNHANES V (2010–2012), and we analyzed the 2010–2011 survey cycles. KNHANES is a cross-sectional study initiated in 1998 to assess the health status of the Korean population. Administered by the Korea Disease Control and Prevention Agency (KDCA), the survey annually encompasses approximately 10,000 participants selected from 3840 households across 192 regions in South Korea. Utilizing a two-stage sampling approach involving stratification, clustering, and weighting, KNHANES, when adjusted for sampling weights, is representative of the entire Korean population (N = 46,286,503), and the details of calculation were introduced in previous research [8,9,10,11].

**Figure 1:**
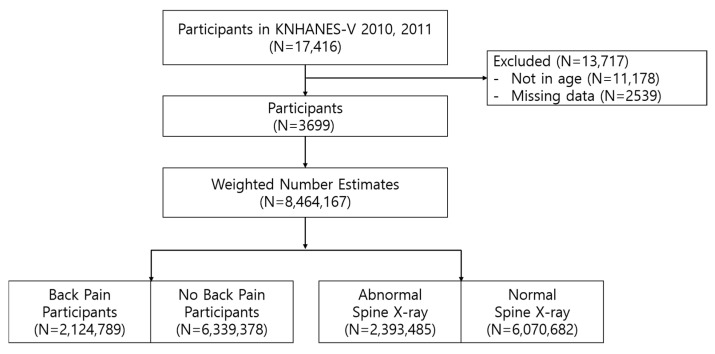
Study design.

Figure 1 outlines the participant selection process, which commenced with 17,416 participants from KNHANES 2010–2011. Exclusions included individuals without lumbar spine radiographs (N = 2539) and those aged under 50 or over 80 years (N = 11,178). Lumbar spine radiographs in KNHANES are obtained only in participants aged 50 years or older, and participants aged 80 years and above were excluded because they were aggregated into a single age category in the survey, precluding precise age-stratified analyses. As KNHANES relies on self-reported questionnaire data, participants with incomplete responses to key variables or clearly implausible entries (e.g., missing age or implausible anthropometric values) were excluded during data cleaning. Consequently, data from 3699 participants aged 50–79 years were included in the final analysis. To ensure national representativeness of Korean adults aged 50–79 years, complex sampling weights were applied, yielding a weighted population estimate of 8,464,167 individuals. These weighted estimates were used to describe population-level patterns and were divided into LBP and non-LBP groups.

Our analysis includes health examination data, reports of LBP, and radiographic lumbar spondylosis based on K–L grades, aiming to elucidate the epidemiological patterns of LBP and identify associated factors within the Korean population. Additionally, participants were subdivided into those with radiographic lumbar spondylosis (K–L grade ≥ 2) and those without radiographic spondylosis [12,13,14].

### 2.2. Characteristics

**Figure 2:**
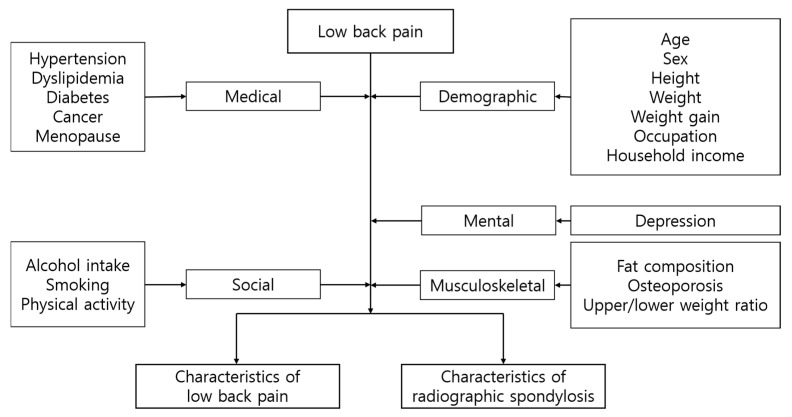
Diagram of multiple types of characteristics assessed in this study.

Figure 2 depicts the parameters employed in our study. Medical and mental factors encompass diagnosed conditions derived from health surveys administered by trained personnel. The KNHANES captures prevalent diagnoses such as hypertension, dyslipidemia, and diabetes. Furthermore, our investigation includes depressive mood as an associated factor, characterized by a sustained depressive mood lasting more than 2 weeks.

Musculoskeletal factors, including body fat compositions measured by dual-energy X-ray absorptiometry (DEXA), were gathered at the mobile examination center. DEXA provides estimates of fat mass and lean mass, offering insights into total and regional percentages of fat and muscle composition. KNHANES datasets encompassed whole-body DEXA measurements of bone mineral density, osteoporosis diagnosis, upper/lower extremity weight ratio, and fat percentage (fat mass/total mass × 100) [15].

Furthermore, this study evaluated social characteristics through questionnaires across three domains: smoking, alcohol intake, and physical activity, all of which directly impact an individual’s health status. Smoking was quantified as cumulative exposure over an individual’s lifetime, measured in pack-years. Pack-years, previously used as a representation of smoking amount, were calculated by multiplying the number of packs of cigarettes smoked per day by the total number of years a person has been smoking. Similarly, alcohol intake was quantified as the weekly average number of bottles of locally popular distilled liquor (360 mL per bottle, ~16% alcohol by volume). Physical activity was computed based on parameters from KNHANES questionnaires and expressed as metabolic equivalents (METs), reflecting the intensity-weighted sum of reported physical activities. A one-unit increase in MET represents a minimal incremental change in overall activity level derived from questionnaire responses rather than a discrete behavioral change [16].

The assessment of spine X-ray radiographs involved the application of the K-L grade. K–L grades were assigned based on the overall impression of degenerative changes across the lumbar spine rather than at individual vertebral levels. Two independent radiologists evaluated the X-rays, and in cases where a one-grade discrepancy existed, the higher grade was considered valid. Discrepancies exceeding one grade underwent thorough review by a third radiologist, and the grade aligned with the third assessment was adopted [9]. Grades 2 to 4 were operationally defined as radiographic lumbar spondylosis, consistent with prior epidemiologic studies using KNHANES data [9,11]. The presence of spine-related pain, known as LBP, was determined through a survey inquiring about the experience of LBP, characterized by the presence of pain in the low back lasting for at least 30 days within the preceding three months. The non-LBP group was defined as participants who reported no low back pain lasting for 30 days or longer during the preceding three months. This definition captures subacute-to-chronic low back pain and has been used in prior KNHANES-based epidemiologic studies. Although alternative duration thresholds are used in other international studies, the present definition was selected to balance recall reliability and clinical relevance within the structure of the KNHANES questionnaire [11,14].

### 2.3. Statistical Analysis

#### 2.3.1. Descriptive Analysis

The baseline characteristics of participants were evaluated separately for male and female groups to identify differences in characteristics between the sexes. A comparable analysis was carried out by stratifying participants into age groups: 50–59, 60–69, and 70–79. Variables such as weight, reported as mean and standard deviation, were subjected to a Student’s *t*-test for comparison. Categorical variables, such as radiographic K-L grade, were presented as percentages and numerical values, and comparisons were made using the chi-squared test. All variables were assessed for normality, and if normality was not met, non-parametric tests, such as the Mann–Whitney U test, were employed for each respective variable. Individual normality test results were not reported, as the analyses were descriptive in nature and focused on group-level patterns rather than formal distributional inference.

#### 2.3.2. Multivariate Logistic Regression

To explore factors associated with LBP, we fitted multivariable logistic regression models, as used in similar studies. This study computed odds ratios (OR) and their corresponding 95% confidence intervals (CI). Additionally, the Variance Inflation Factor (VIF) was calculated to assess the presence of multicollinearity among variables, and VIF < 10 was accepted as no multicollinearity.

#### 2.3.3. Propensity Score Matching

Previous research has indicated that age, sex, and the K–L grade of spine radiographs are significant objective factors associated with the likelihood of experiencing LBP. To explore patterns of association with LBP under comparable objective conditions, we performed sex-stratified propensity score (PS) matching; specifically, the population was stratified by sex, and within each sex, participants with and without LBP were matched on age and K–L grade of spine radiographs.

PS matching was used as an illustrative approach to compare participants under similar age and radiographic conditions, without implying causal control of confounding [11,17]. The PS denotes the conditional probability of belonging to the LBP group given age and lumbar radiographic grade within each sex and is commonly utilized in social science and medical analyses [18,19]. Participants with similar propensity scores were matched using the nearest neighbor method with a predefined PS caliper. A 1:1 matching ratio was applied within each sex to enhance comparability between the LBP and non-LBP groups. The standardized mean difference (SMD) was used to evaluate matching quality, with SMD < 0.1 indicating well-balanced matched pairs. Following matching, participant characteristics were compared using Student’s *t*-test for continuous variables and the chi-squared test for categorical variables.

All analyses were performed using R version 4.2.2 on the Windows 10 operating system and various R libraries, including ‘dplyr’ (version 1.0.10), ‘moonBook’ (version 0.3.1), and related packages. The level of significance was set at *p* < 0.05.

## 3. Results

### 3.1. Descriptive Analysis

The descriptive analysis summarized sex-specific characteristics (Table 1). In the survey-weighted Korean population, 25.1% reported LBP and 28.3% demonstrated radiographic lumbar spondylosis defined as K–L grade ≥ 2. Female participants exhibited a higher prevalence of LBP and radiographic lumbar spondylosis than male participants. Sex-specific differences in other epidemiologic factors were analyzed subsequently.

The age-stratified analysis classified participants into 50–59, 60–69, and 70–79-year groups (Table 2). In weighted estimates, the prevalence of LBP increased progressively with age and was paralleled by a higher proportion of participants with radiographic lumbar spondylosis. Age-related differences in body composition, occupational status, and socioeconomic measures are summarized in Table 2.

### 3.2. Multivariable Logistic Regression to Identify Factors Associated with LBP

Table 3 shows the result of multivariate logistic regression to investigate associated factors for LBP in Korean men. Notably, radiographic lumbar spondylosis was correlated with increased reports of LBP. Additionally, LBP was associated with aging, greater height, recent weight gain, and a higher upper/lower extremity weight ratio. Individuals engaged in white-collar occupations and those with lower household incomes were more likely to experience low back pain. Moreover, diagnoses of diabetes, cancer, osteoporosis, and depressive mood were linked with LBP. Social factors such as higher alcohol intake, increased smoking, and greater physical activity levels were also associated with an elevated reporting of LBP. The VIF for all factors was less than 10, indicating the absence of multicollinearity.

Table 4 presents factors associated with LBP in Korean women. LBP was associated with radiographic lumbar spondylosis and with older age, higher body weight, recent weight gain, and a lower upper-to-lower extremity weight ratio. In addition, hypertension, dyslipidemia, and osteoporosis were associated with higher odds of LBP. Social and behavioral factors, including greater alcohol intake and higher physical activity levels, were also associated with LBP. Moreover, blue-collar occupation and lower household income were associated with LBP. The VIF was <10 for all variables, suggesting no problematic multicollinearity.

### 3.3. PS Matching for Exploring Factors of Back Pain When in the Same Objective Condition

To assess factors associated with LBP independent of age and radiographic status, we performed PS matching on age and severity of radiographic lumbar spondylosis. After 1:1 PS matching, SMDs for the matching variables were <0.1, indicating good balance between groups. In the matched analysis, weighted population estimates corresponding to men with and without LBP were compared descriptively (Table 5). In the matched cohort, diagnoses of diabetes and cancer, depressive symptoms, osteoporosis, and a higher upper-to-lower extremity weight ratio were more common among participants reporting LBP. Occupational (blue-collar work) and socioeconomic (lower household income) factors were also associated with LBP, and higher levels of alcohol consumption, smoking exposure, and physical activity correlated positively with LBP (all *p* < 0.001).

We identified 1,473,767 Korean women who reported experiencing LBP and an equivalent number of participants who did not report LBP, matched for age and severity of radiographic lumbar spondylosis (Table 6).

The presence of hypertension, dyslipidemia, and diabetes diagnoses exhibited associations with LBP (*p* < 0.001). Additionally, depressive symptoms were correlated with the reporting of LBP. Osteoporosis and a lower upper/lower extremity weight ratio were also identified as factors more likely to be associated with LBP. Engagement in blue-collar occupations and lower household income were further linked to LBP. In terms of social factors, a positive correlation was observed between high alcohol intake, smoking frequency, and physical activity levels with the occurrence of LBP.

## 4. Discussion

In survey-weighted analyses from KNHANES V (unweighted n = 3699; weighted population estimate = 8,464,167 adults aged 50–79 years), we characterized the epidemiology of LBP among Korean adults aged 50–79 years. In weighted estimates, 25.1% reported LBP, and 28.3% demonstrated radiographic lumbar spondylosis, defined as a high K–L grade on spine radiographs.

The relationship between radiologic findings and the clinical experience of LBP remains incompletely understood. Twin studies from the Twin Spine Study indicate that genetic factors substantially contribute to inter-individual differences in spinal degeneration, often outweighing occupational or activity-related exposures [20]. Furthermore, the etiology of LBP is heterogeneous; symptoms may originate from discogenic sources (e.g., internal disc disruption or annulus fibrosus pathology) or from non-discogenic mechanisms (e.g., nerve-root or dorsal root ganglion irritation, facet joint pathology, or ligamentous disease), and the precise pain generator is frequently difficult to localize [21,22]. Given this multifactorial and often poorly localized nature of LBP—and the imperfect concordance between imaging findings and symptoms—we prioritized clinically accessible, routinely obtainable measures, specifically patient age and standard spine radiography graded by the K–L scale, rather than relying on resource-intensive modalities such as MRI. This decision was supported by evidence that many MRI-detected degenerative changes in the spine are poorly correlated with symptoms and may offer limited clinical value for identifying LBP [6]. Therefore, rather than relying on resource-intensive MRI, we used K–L grading on plain radiographs as an objective marker of radiographic lumbar spondylosis and controlled for age and sex—intuitive, easily obtainable patient characteristics in routine clinical settings. Furthermore, by matching participants on age, sex, and radiographic status (K–L grade), we evaluated factors associated with LBP under comparable radiographic and demographic conditions. This design provides a distinct perspective on LBP by allowing us to examine which additional factors, beyond the most intuitive determinants (age, sex, and radiographic severity on plain radiographs), are associated with LBP.

Descriptive analysis revealed the distinct characteristics by sex. Previous studies have emphasized the role of estrogen in understanding sex-based differences in joint pain. In particular, female participants tend to experience an increase in the occurrence of osteoarthritis at multiple sites around the menopausal transition [23]. Multiple studies have also suggested that estrogen may exert protective effects against reactive oxygen species and pro-inflammatory cytokines within the joint space, indicating a complex role in not only the onset but also the progression of chronic spinal changes [24,25]. Considering the participants in our study were aged 50 years and above, about 90% of female participants had already undergone menopause. The decline in estrogen and related hormonal changes may contribute to higher BMI, greater adiposity, and a higher prevalence of osteoporosis among women in this age range.

In survey-weighted analyses, the extremely narrow confidence intervals and statistical associations with small effect sizes observed in this study likely reflect the large effective population size implied by survey weighting, together with incomplete incorporation of complex survey design features in variance estimation, rather than strong individual-level effects. Accordingly, statistical significance in this study should not be interpreted as clinical importance or precise population-level inference. These findings are best viewed as identifying population-level correlates of LBP rather than determinants with large individual effects. The matching analysis was intended as an exploratory approach to illustrate patterns of association under comparable age and radiographic conditions rather than to support causal inference. Given the cross-sectional design and the complexity of survey-weighted data, these results should be interpreted as descriptive and hypothesis-generating rather than as providing definitive causal estimates. Therefore, confidence intervals and *p*-values presented in this study are provided for descriptive completeness only and should not be interpreted as valid inferential statistics for population-level conclusions.

Several covariates showed sex-specific directionality in the multivariable models (e.g., opposite associations for hypertension and dyslipidemia, and an apparently protective association of smoking in women). Such discordant patterns may reflect true effect modification by sex, but may also arise from differences in exposure distribution, residual confounding structures, and measurement properties across sexes. In particular, self-reported behaviors and diagnoses are susceptible to differential misclassification, which can bias odds ratios in unpredictable directions [26].

This study evaluated multiple factors for LBP, several of which may be linked to inflammatory pathways [27]. Diabetes, a metabolic disorder characterized by elevated circulating free fatty acids and a pro-inflammatory state, may contribute to vascular dysfunction and calcification, potentially impairing nutrient delivery to the intervertebral disc and increasing susceptibility to degeneration and pain, thereby contributing to LBP [28]. Moreover, obesity, which commonly co-occurs with diabetes, can further exacerbate systemic inflammation. Consistent with prior reports, we observed an association between recent weight gain and LBP [29]. Alcohol intake was associated with LBP in multiple previous studies, showing that circulating pro-inflammatory cytokines produced by alcohol intake elevated inflammatory status level and aggravated LBP [30]. In addition, fat mass and its distribution are associated with LBP intensity and disability, and systemic metabolic factors related to adiposity may play an important role in the pathogenesis of LBP [31,32].

The upper-to-lower extremity mass ratio may reflect regional body-composition distribution; its association with LBP may relate to adiposity distribution and reduced trunk skeletal muscle mass, both of which have been linked to LBP and impaired lumbar function in prior studies, although evidence regarding the magnitude and direction of these associations remains mixed [19,33]. Because our study was cross-sectional, causality cannot be inferred; however, our findings suggest that body-mass distribution may be associated with LBP, and further longitudinal studies are warranted to clarify this relationship. Osteoporosis and high levels of physical activity were also associated with increased LBP. One potential explanation is that LBP can arise from cumulative micro-injuries to spinal joints and supporting structures [34]. Osteoporosis may reduce structural resilience and increase susceptibility to injury from repetitive loading [35]. Likewise, high levels of physical activity may increase exposure to mechanical strain and injury, contributing to LBP [36]. However, this interpretation should be considered alongside evidence suggesting that genetic background may account for a large proportion of inter-individual variability in degenerative spinal changes and related symptoms, potentially attenuating the apparent contribution of activity- or occupation-related exposures [20]. Alcohol intake and physical activity were quantified using standardized units; however, the unit increments—particularly for physical activity measured in METs—represent relatively small changes in exposure. Therefore, the associated odds ratios should be interpreted as modest, descriptive associations rather than clinically meaningful effects at the individual level.

Prior studies suggest that depressive mood can influence somatic pain perception [37,38]. Consistent with this framework, individuals with depressive mood may report more severe LBP even in the absence of marked radiographic abnormalities, underscoring the importance of psychosocial factors in the management of musculoskeletal pain.

Lower household income was associated with LBP. The link between low socioeconomic status and an increased prevalence of LBP has been extensively investigated in previous research. Studies conducted in the United States have revealed that lower socioeconomic status, including a higher poverty rate, was associated with an elevated osteoarthritic index [39,40]. Limited access to healthcare and gaps in insurance coverage have been proposed as contributing mechanisms in some settings. In Korea, however, healthcare coverage is near-universal through the national health insurance system, with additional support programs for low-income citizens [41]. Therefore, alternative explanations should be considered, including differences in occupational exposures, health literacy, and healthcare-seeking behavior [42].

This study has several limitations. First, KNHANES is cross-sectional; therefore, our findings reflect associations rather than causal relationships, and future prospective studies are needed to clarify the temporal links between factors and LBP. Second, the dataset lacks detailed musculoskeletal diagnoses, limiting our ability to distinguish specific etiologies of LBP attributable to conditions such as facet joint osteoarthritis, spinal stenosis, disc protrusion, spondylolisthesis, or spinal metastasis; diagnosis-based analyses would provide a more granular epidemiologic understanding. Third, our analytic sample was restricted to participants aged ≥ 50 years because spine radiographs are obtained only in this age group in KNHANES, and we excluded those aged ≥ 80 years because they were aggregated into a single category, precluding precise age-stratified evaluation. Although inclusion of all age groups might have yielded broader epidemiologic insight, these age restrictions were necessary given the structure of the available data. Finally, KNHANES uses a complex, stratified, multistage cluster sampling design, and sampling weights are provided to obtain nationally representative point estimates. Accordingly, population counts presented in our tables should be interpreted as weighted population estimates rather than actual sample sizes. Because variance estimation in complex surveys depends on strata and cluster information, analyses that do not fully incorporate the survey design may underestimate standard errors and yield overly small *p*-values [43]. In addition, propensity score methods in complex survey data require careful handling of weights and design features, and ignoring these components can compromise population-level inference [44]. Therefore, our findings should be interpreted as hypothesis-generating associations, and future work should re-estimate key results using design-based survey procedures and survey-aware propensity score approaches to assess robustness.

From a clinical perspective, the present findings suggest that LBP reflects a multidimensional health state influenced by psychosocial, socioeconomic, metabolic, and musculoskeletal factors, even among individuals with similar radiographic findings. This supports a broader clinical assessment of LBP that extends beyond imaging findings alone.

## 5. Conclusions

In this nationally representative study of Korean adults aged 50–79 years, LBP affected approximately one-quarter of the population and was associated with radiographic lumbar spondylosis. However, even after matching for age and lumbar X-ray grade, LBP remained associated with depressive mood, osteoporosis, lower socioeconomic status, and lifestyle-related factors, suggesting that age and radiographic severity alone may not fully explain the presence of LBP. Although causal relationships cannot be inferred from this cross-sectional study, these results highlight consistent associations between LBP and psychosocial, systemic, and socioeconomic factors beyond lumbar radiographic severity. These findings should be interpreted as hypothesis-generating and as supporting a multidimensional approach to LBP assessment in both clinical and public health contexts.

## Figures and Tables

**Table 1:** Descriptive analysis of participants by sex.

	Total (N = 8,464,167)	Male (N = 3,987,665)	Female (N = 4,476,502)
Age	61.0 ± 8.2	60.4 ± 7.9 *	61.4 ± 8.5 *
Height (cm)	160.4 ± 8.8	167.5 ± 5.9 *	154.1 ± 5.7 *
Weight (kg)	62.1 ± 10.0	66.8 ± 9.5 *	57.9 ± 8.5 *
BMI (kg/m^2^)	24.1 ± 3.1	23.8 ± 2.8 *	24.3 ± 3.2 *
Recent weight gain in 1 year	10.8%	7.8% *	13.5% *
Occupation type (Blue collar)	46.2%	57.6% *	35.9% *
Household income (top 50%)	44.8%	50.1% *	40.1% *
Diagnosis of Hypertension	37.5%	33.7% *	40.9% *
Diagnosis of Dyslipidemia	16.9%	13.2% *	20.1% *
Diagnosis of Diabetes	14.0%	15.4% *	12.7% *
Diagnosis of Cancer	4.7%	3.5% *	5.8% *
Menopause	48.4%	0 *	91.5% *
Depressive Mood (>14 days)	15.3%	10.5% *	19.5% *
Alcohol intake	0.8 ± 1.6	1.6 ± 2.0 *	0.2 ± 0.6 *
Smoking amount	11.9 ± 18.9	24.0 ± 21.1 *	1.1 ± 5.8 *
Physical activity	268.7 ± 485.6	298.0 ± 497.6 *	242.6 ± 473.2 *
Fat composition (%)	29.2 ± 8.1	22.6 ± 5.1 *	35.0 ± 5.3 *
Upper/Lower extremity weight ratio	0.60 ± 0.05	0.61 ± 0.05 *	0.60 ± 0.05 *
Diagnosis of Osteoporosis	9.4%	1.3% *	16.5% *
Low back pain	25.1%	14.1% *	34.9% *
Radiographic lumbar spondylosis	28.3%	24.7% *	31.5% *

* denotes *p* < 0.001 in comparison between sexes. BMI; Body mass index. Radiographic lumbar spondylosis; Lumbar spine K-L grade ≥ 2.

**Table 2:** Descriptive analysis of participants by age groups.

	Age Group 50–59 (N = 4,334,678)	Age Group 60–69 (N = 2,505,196)	Age Group 70–79 (N = 1,624,293)
Age	54.1 ± 2.8 *	64.4 ± 2.9 *	73.9 ± 2.8 *
Sex (Female)	50.7% *	52.0% *	60.2% *
Height (cm)	162.2 ± 8.5 *	160.1 ± 8.3 *	156.1 ± 8.8 *
Weight (kg)	63.6 ± 9.8 *	62.1 ± 9.8 *	58.0 ± 9.6 *
BMI (kg/m^2^)	24.1 ± 2.9 *	24.2 ± 3.1 *	23.8 ± 3.3 *
Recent weight gain in 1 year	14.3% *	7.9% *	5.9% *
Occupation type (Blue collar)	51.6% *	46.9% *	30.4% *
Household income (top 50%)	60.5% *	34.3% *	19.1% *
Diagnosis of Hypertension	27.1% *	44.6% *	54.4% *
Diagnosis of Dyslipidemia	15.1% *	21.1% *	15.2% *
Diagnosis of Diabetes	10.5% *	16.0% *	20.1% *
Diagnosis of Cancer	3.7% *	5.3% *	6.6% *
Menopause	41.9% *	52.0% *	60.2% *
Depressive Mood (>14 days)	16.2% *	14.1% *	14.6% *
Alcohol intake	1.0 ± 1.8 *	0.7 ± 1.5 *	0.5 ± 1.3 *
Smoking amount	11.6 ± 16.8 *	12.1 ± 20.8 *	12.3 ± 21.0 *
Physical activity	312.8 ± 506.2 *	244.3 ± 453.9 *	188.4 ± 463.2 *
Fat composition (%)	28.7 ± 8.0 *	29.4 ± 8.2 *	30.2 ± 8.2 *
Upper/Lower extremity weight ratio	0.60 ± 0.05 *	0.61 ± 0.05 *	0.62 ± 0.05 *
Diagnosis of Osteoporosis	4.8% *	12.5% *	16.8% *
Low back pain	17.9% *	28.8% *	38.6% *
Radiographic lumbar spondylosis	15.9% *	31.5% *	56.4% *

* denotes *p* < 0.001 in comparison among age groups. BMI; Body mass index. Radiographic lumbar spondylosis; Lumbar spine K-L grade ≥ 2.

**Table 3:** Associated factors for LBP in Korean men.

Factor	Coefficient	OR (95% CI)	*p*-Value
Age	0.0181	1.018 (1.017–1.018)	<0.001
Height (cm)	0.0371	1.038 (1.037–1.038)	<0.001
Weight (kg)	−0.0123	0.987 (0.987–0.988)	<0.001
Recent weight gain in 1 year	0.1567	1.169 (1.156–1.182)	<0.001
Occupation type (Blue collar)	−0.1165	0.889 (0.884–0.895)	<0.001
Household income (top 50%)	−0.3638	0.694 (0.690–0.699)	<0.001
Diagnosis of Hypertension	−0.3845	0.680 (0.676–0.685)	<0.001
Diagnosis of Dyslipidemia	−0.0403	0.960 (0.951–0.969)	<0.001
Diagnosis of Diabetes	0.2222	1.248 (1.238–1.259)	<0.001
Diagnosis of Cancer	0.6596	1.934 (1.908–1.960)	<0.001
Depressive Mood (>14 days)	0.6889	1.991 (1.975–2.007)	<0.001
Alcohol intake	0.0566	1.058 (1.056–1.059)	<0.001
Smoking amount	0.0049	1.004 (1.004–1.005)	<0.001
Physical activity	0.0003	1.001 (1.000–1.001)	<0.001
Fat composition (%)	−0.0055	0.994 (0.993–0.995)	<0.001
Upper/Lower extremity weight ratio	0.5046	1.656 (1.549–1.770)	<0.001
Diagnosis of Osteoporosis	0.6333	1.883 (1.844–1.923)	<0.001
Radiographic lumbar spondylosis	0.6625	1.939 (1.927–1.952)	<0.001
Intercept	−9.0581		

Radiographic lumbar spondylosis; Lumbar spine K-L grade ≥ 2.

**Table 4:** Associated factors for LBP in Korean women.

Factor	Coefficient	OR (95% CI)	*p*-Value
Age	0.0456	1.046 (1.046–1.047)	<0.001
Height (cm)	−0.0028	0.997 (0.996–0.997)	<0.001
Weight (kg)	0.0107	1.010 (1.010–1.011)	<0.001
Recent weight gain in 1 year	0.3411	1.406 (1.397–1.415)	<0.001
Occupation type (Blue collar)	0.0531	1.054 (1.049–1.059)	<0.001
Household income (top 50%)	−0.3398	0.711 (0.708–0.715)	<0.001
Diagnosis of Hypertension	0.1672	1.182 (1.176–1.187)	<0.001
Diagnosis of Dyslipidemia	0.4005	1.492 (1.484–1.500)	<0.001
Diagnosis of Diabetes	0.0039	1.003 (0.997–1.010)	0.240
Diagnosis of Cancer	0.0028	1.002 (0.993–1.011)	0.533
Menopause	−0.3285	0.719 (0.713–0.726)	<0.001
Depressive Mood (>14 days)	0.5780	1.782 (1.773–1.791)	<0.001
Alcohol intake	0.1450	1.156 (1.151–1.160)	<0.001
Smoking amount	−0.0065	0.993 (0.993–0.994)	<0.001
Physical activity	0.0003	1.001 (1.000–1.001)	<0.001
Fat composition (%)	−0.0259	0.974 (0.973–0.974)	<0.001
Upper/Lower extremity weight ratio	−0.3525	0.702 (0.672–0.734)	<0.001
Diagnosis of Osteoporosis	0.7775	2.176 (2.164–2.188)	<0.001
Radiographic lumbar spondylosis	0.4966	1.643 (1.635–1.651)	<0.001
Intercept	−3.1607		

Radiographic lumbar spondylosis; Lumbar spine K-L grade ≥ 2.

**Table 5:** Associated Factors for Low Back Pain in Korean Men under Comparable Age and Radiographic Conditions (Weighted Population Estimates).

Male Participants	No LBP (N = 561,072)	Reported LBP (N = 561,072)
Age	62.0 ± 8.0	62.0 ± 8.0
Radiographic lumbar spondylosis	37.9%	37.9%
The SMD of age and radiographic lumbar spondylosis < 0.1: Well-matched		
Height (cm)	167.3 ± 5.9 *	167.7 ± 5.6 *
Weight (kg)	66.4 ± 9.8 *	65.4 ± 9.1 *
BMI (kg/m^2^)	23.7 ± 2.9 *	23.2 ± 2.7 *
Recent weight gain in 1 year	5.9% *	7.9% *
Occupation type (Blue collar)	53.4% *	57.4% *
Household income (top 50%)	48.3% *	38.2% *
Diagnosis of Hypertension	38.4% *	28.1% *
Diagnosis of Dyslipidemia	14.0% *	11.0% *
Diagnosis of Diabetes	14.6% *	17.2% *
Diagnosis of Cancer	1.5% *	5.8% *
Depressive Mood (>14 days)	10.0% *	17.6% *
Alcohol intake	1.4 ± 2.0 *	1.8 ± 2.2 *
Smoking amount	25.2 ± 22.6 *	27.9 ± 22.6 *
Physical activity	253.3 ± 401.9 *	340.1 ± 647.4 *
Fat composition (%)	22.7 ± 5.0 *	21.9 ± 5.3 *
Upper/Lower extremity weight ratio	0.61 ± 0.05 *	0.62 ± 0.04 *
Diagnosis of Osteoporosis	0.8% *	2.3% *

* denotes descriptive comparisons only. *p*-values are reported for completeness and should not be interpreted as inferential statistics. All values represent weighted population estimates derived from KNHANES sampling weights; actual analytic sample sizes were substantially smaller. Radiographic lumbar spondylosis; Lumbar spine K-L grade ≥ 2. All values represent weighted population estimates derived from KNHANES sampling weights. Actual analytic sample sizes were substantially smaller.

**Table 6:** Associated Factors for Low Back Pain in Korean Women under Comparable Age and Radiographic Conditions (Weighted Population Estimates).

Female Participants	No LBP (N = 1,473,767)	Reported LBP (N = 1,473,767)
Age	63.7 ± 8.4	63.7 ± 8.5
Radiographic lumbar spondylosis	39.2%	39.2%
The SMD of age and radiographic lumbar spondylosis < 0.1: Well-matched		
Height (cm)	153.6 ± 5.5	153.6 ± 5.8
Weight (kg)	57.6 ± 7.9 *	57.8 ± 9.4 *
BMI (kg/m^2^)	24.4 ± 3.1 *	24.4 ± 3.5 *
Recent weight gain in 1 year	12.1% *	13.5 *
Occupation type (Blue collar)	31.0% *	39.7% *
Household income (top 50%)	38.6% *	30.5% *
Diagnosis of Hypertension	45.3% *	47.5% *
Diagnosis of Dyslipidemia	20.6% *	24.4% *
Diagnosis of Diabetes	14.8% *	15.0% *
Diagnosis of Cancer	6.4% *	6.0% *
Menopause	95.9% *	93.5% *
Depressive Mood (>14 days)	15.5% *	25.5% *
Alcohol intake	0.1 ± 0.4 *	0.2 ± 0.7 *
Smoking amount	0.9 ± 5.0 *	1.1 ± 6.3 *
Physical activity	212.5 ± 405.9 *	274.8 ± 570.0 *
Fat composition (%)	35.5 ± 5.0 *	34.8 ± 5.8 *
Upper/Lower extremity weight ratio	0.61 ± 0.05 *	0.60 ± 0.05 *
Diagnosis of Osteoporosis	14.9% *	25.3% *

* denotes descriptive comparisons only. *p*-values are reported for completeness and should not be interpreted as inferential statistics. All values represent weighted population estimates derived from KNHANES sampling weights; actual analytic sample sizes were substantially smaller. Radiographic lumbar spondylosis; Lumbar spine K-L grade ≥ 2. All values represent weighted population estimates derived from KNHANES sampling weights. Actual analytic sample sizes were substantially smaller.

## Data Availability

The original data presented in the study are openly available at the following website: https://knhanes.kdca.go.kr/knhanes/main.do (accessed on 12 December 2025).

## Abbreviations

The following abbreviations are used in this manuscript:

BMI	Body Mass Index
DEXA	Dual-Energy X-ray Absorptiometry
KNHANES	Korea National Health and Nutrition Examination Survey
K–L	Kellgren–Lawrence
LBP	Low Back Pain
PS	Propensity Score
SMD	Standardized Mean Difference
VIF	Variance Inflation Factor
